# Locus coeruleus cellular and molecular pathology during the progression of Alzheimer’s disease

**DOI:** 10.1186/s40478-017-0411-2

**Published:** 2017-01-21

**Authors:** Sarah C. Kelly, Bin He, Sylvia E. Perez, Stephen D. Ginsberg, Elliott J. Mufson, Scott E. Counts

**Affiliations:** 1 Department of Translational Science and Molecular Medicine, Michigan State University, 333 Bostwick Ave NE, Grand Rapids, MI 49503 USA; 2 Cell and Molecular Biology Program, Michigan State University, East Lansing, MI USA; 3 Department of Family Medicine, Michigan State University, East Lansing, MI USA; 4 Hauenstein Neurosciences Center, Mercy Health Saint Mary’s Hospital, Grand Rapids, MI USA; 5 Department of Neurobiology, Barrow Neurological Institute, Phoenix, AZ USA; 6 Center for Dementia Research, Nathan Kline Institute, Orangeburg, NY USA; 7 Department of Psychiatry, New York University Langone School of Medicine, New York, NY USA; 8 Department of Neuroscience and Physiology, New York University Langone School of Medicine, New York, NY USA

**Keywords:** Locus coeruleus, Norepinephrine, Mild cognitive impairment, Alzheimer’s disease, Neurodegeneration, Gene expression

## Abstract

A major feature of Alzheimer’s disease (AD) is the loss of noradrenergic locus coeruleus (LC) projection neurons that mediate attention, memory, and arousal. However, the extent to which the LC projection system degenerates during the initial stages of AD is still under investigation. To address this question, we performed tyrosine hydroxylase (TH) immunohistochemistry and unbiased stereology of noradrenergic LC neurons in tissue harvested postmortem from subjects who died with a clinical diagnosis of no cognitive impairment (NCI), amnestic mild cognitive impairment (aMCI, a putative prodromal AD stage), or mild/moderate AD. Stereologic estimates of total LC neuron number revealed a 30% loss during the transition from NCI to aMCI, with an additional 25% loss of LC neurons in AD. Decreases in noradrenergic LC neuron number were significantly associated with worsening antemortem global cognitive function as well as poorer performance on neuropsychological tests of episodic memory, semantic memory, working memory, perceptual speed, and visuospatial ability. Reduced LC neuron numbers were also associated with increased postmortem neuropathology. To examine the cellular and molecular pathogenic processes underlying LC neurodegeneration in aMCI, we performed single population microarray analysis. These studies revealed significant reductions in select functional classes of mRNAs regulating mitochondrial respiration, redox homeostasis, and neuritic structural plasticity in neurons accessed from both aMCI and AD subjects compared to NCI. Specific gene expression levels within these functional classes were also associated with global cognitive deterioration and neuropathological burden. Taken together, these observations suggest that noradrenergic LC cellular and molecular pathology is a prominent feature of prodromal disease that contributes to cognitive dysfunction. Moreover, they lend support to a rational basis for targeting LC neuroprotection as a disease modifying strategy.

**Electronic supplementary material:**

The online version of this article (doi:10.1186/s40478-017-0411-2) contains supplementary material, which is available to authorized users.

## Introduction

Sporadic Alzheimer’s disease (AD) is believed to have an extensive preclinical stage since neuropathological examinations of older people with a clinical diagnosis of no or mild cognitive impairment (MCI; a putative prodromal stage of AD) consistently reveal similar pathological signatures to those with frank AD [2, 77]. Thus, identifying events that occur in the preclinical stages of AD will be essential for therapeutic target identification within a disease-modifying window. In this regard, noradrenergic neurons of the nucleus locus coeruleus (LC) projection system, which provide the primary source of norepinephrine (NE) to the forebrain, mediate memory and attention [16, 105] and degenerate in advanced AD [19, 24, 36, 75, 86, 119], yet there is evidence for the involvement of this system earlier in the disease process. For instance, neurofibrillary tangle (NFT) deposition in the LC has been noted in aged control and cognitively impaired subjects and LC NFTs correlate with global cognition [55], in line with reduced NE levels in the hippocampus and cortex in AD [1, 75, 94]. Moreover, it has been suggested that the LC is an initial site of NFT formation in young adults and during aging, thus serving as a potential nidus for NFT propagation during disease progression [21, 55].

Reductions in LC neuron number are also associated with increased cortical amyloid plaque and NFT loads [18, 108] and correlate better with onset and duration of AD than cholinergic nucleus basalis degeneration [48, 76, 124]. More recently, morphometric studies have shown that 1) the volume and number of total neurons in the LC undergo a step-wise reduction with successive Braak stage [110], and 2) there is a significant loss of neuromelanin-containing LC neurons in individuals meeting the clinical pathologic criteria for MCI [e.g., clinical dementia rating (CDR) = 0.5 with low to intermediate AD pathology] [7]. In the present study, we expanded on these observations by using tyrosine hydroxylase (TH) immunohistochemistry and unbiased stereology to investigate the extent of noradrenergic LC neuron loss in postmortem samples obtained from subjects who received an antemortem clinical diagnosis of no cognitive impairment (NCI), amnestic MCI (aMCI; the MCI subtype most likely to convert to frank AD [96, 121, 122]), or mild/moderate AD. TH-immunoreactive (-ir) LC neuron numbers were evaluated with respect to both antemortem neuropsychological test scores and postmortem pathological diagnostic criteria.

While morphometric studies of LC cell loss help to delineate its relative status within the spatiotemporal pattern of selective neuronal vulnerability in AD [7, 108, 124], the molecular mechanisms underlying LC neurodegeneration during disease progression have yet to be fully understood. To gain a better understanding of potential molecular mechanisms driving LC neuronal dysfunction during the onset of AD, we combined single neuron RNA amplification strategies with custom-designed microarrays to analyze differential gene expression patterns of individual TH-ir LC neurons accrued from the same NCI, aMCI, and AD cases.

## Materials and methods

### Subjects and clinical neuropathologic assessments

Brainstems from de-identified subjects who died with an antemortem clinical diagnosis of NCI (*n* = 11), aMCI (*n* = 10) or mild/moderate AD (*n* = 8) representing both genders were obtained from participants in the Rush Religious Orders Study (RROS), a longitudinal clinical pathologic study of aging and AD in elderly Catholic clergy [14, 87]. The study was determined to be exempt from IRB review by the participating institutions. Although RROS participants are not representative of a community-based sample, the basic neurobiological process underlying cognitive impairment in this cohort is likely the same as in others. For instance, neuropathological studies of MCI show similar results in the RROS [13, 67, 88], Washington University [100], and University of Kentucky [77] cohorts.

Table 1 summarizes the demographic, clinical, and neuropathological characteristics of the subjects examined. Details of cognitive evaluations and diagnostic criteria have been extensively published [14, 34, 87, 95]. Briefly, a team of investigators performed annual neuropsychological performance testing including the Mini Mental State Exam (MMSE) and 17 additional neuropsychological tests referable to five cognitive domains: episodic memory (immediate and delayed recall of the East Boston Story and Story A from Logical Memory, Word List Memory, Word List Recall, Word List Recognition), semantic memory (15-item Boston Naming Test, Verbal Fluency, 15-item word reading test), working memory (Digit Span Forward, Digit Span Backward, Digit Ordering), perceptual speed (Symbol Digit Modalities, Number Comparison), and visuospatial abililty (Judgment of Line Orientation, Standard Progressive Matrices). A Global Cognitive Score (GCS), consisting of a composite *z*-score calculated from this test battery, was determined for each participant. A board-certified neurologist with expertise in the evaluation of the elderly made the clinical diagnosis based on impairments in each of the five cognitive domains and a clinical examination. The diagnosis of dementia or AD met recommendations by the joint working group of the National Institute of Neurologic and Communicative Disorders and Stroke/AD and Related Disorders Association (NINCDS/ADRDA) [79]. The aMCI subjects exhibited impairment in episodic memory on neuropsychological testing but did not meet the criteria for AD or dementia. These criteria for aMCI are consistent with those used by others in the field [97].

**Table 1:** Clinical, demographic, and neuropathological characteristics by diagnosis category

	Clinical Diagnosis				
	NCI (*N* = 11)	aMCI (*N* = 10)	AD (*N* = 8)	P-value	Pair-wise comparison
Age (years) at death:					
Mean ± SD	82.3 ± 2.9	85.4 ± 7.2	84.8 ± 2.6	0.3^a^	--
(Range)	(77–86)	(74–96)	(80–88)		
Number (%) of males:	6 (54%)	4 (40%)	4 (50%)	0.5^b^	--
Years of education:					
Mean ± SD	19.1 ± 2.1	17.9 ± 5.2	19.0 ± 2.7	0.9^a^	--
(Range)	(15–22)	(8–23)	(14–22)		
Number (%) with ApoE ε4 allele:	3 (27%)	2 (20%)	3 (38%)	0.2^b^	--
MMSE:					
Mean ± SD	27.9 ± 1.5	26.3 ± 2.3	20.0 ± 4.9	0.0008^a^	NCI > AD
(Range)	(26–30)	(22–30)	(14–27)		
Global Cognitive Score:					
Mean ± SD	0.59 ± 0.3	0.02 ± 0.3	−1.0 ± 0.6	0.0002^a^	NCI > (MCI, AD)
(Range)	(−0.08–0.9)	(−0.53–0.3)	(−1.5– −0.2)		
Post-mortem interval (hours):					
Mean ± SD	4.9 ± 2.0	6.2 ± 5.2	4.0 ± 1.1	0.8^a^	--
(Range)	(2.2–8.5)	(2.0–15.0)	(2.7–5.8)		
Distribution of Braak scores:					
0	0	0	0		
I/II	4	1	0	0.02^a^	NCI < AD
III/IV	6	7	3		
V/VI	1	2	5		
NIA Reagan diagnosis (likelihood of AD):					
No AD	0	0	0		
Low	5	4	0	0.03^a^	(NCI, MCI) < AD
Intermediate	5	4	4		
High	1	2	4		
CERAD diagnosis:					
No AD	3	4	0		
Possible	2	2	0	0.02^a^	(NCI, MCI) < AD
Probable	4	2	3		
Definite	2	2	5		

^a^Kruskal-Wallis test, with Bonferroni correction for multiple comparisons. ^b^Fisher’s exact test, with Bonferroni correction for multiple comparisons

At autopsy, tissue from one hemisphere and brainstem was immersion-fixed in a solution consisting of 4% paraformaldehyde in 0.1 M phosphate buffer (pH 7.2) for 24–72 h at 4 °C followed by cryoprotection, whereas tissue from the opposite hemisphere was frozen at −80 °C [34, 53, 88]. Series of fixed paraffin embedded tissue were prepared for neuropathological evaluation including visualization and quantitation of neocortical and hippocampal amyloid plaques and NFTs using antibodies directed against Aβ peptide (Aβ; 4G8, Covance), tau (PHF1, a gift from Dr. Peter Davies, Hofstra Northwell School of Medicine) [14, 88], as well as thioflavine-S histochemistry and a modified Bielschowsky silver stain. Lewy bodies were revealed using antibodies directed against ubiquitin and α-synuclein. Exclusion criteria for cases selected for this study included evidence of argyrophilic grain disease, frontotemporal dementia, Lewy body disease, mixed dementia, Parkinson’s disease, and stroke. A board certified neuropathologist blinded to the clinical diagnosis performed the neuropathological evaluation. Neuropathological criteria were based on NIA-Reagan, CERAD, and Braak staging [20, 61, 82]. Amyloid burden and apolipoprotein E (ApoE) genotype were determined for each case as described previously [14, 88].

### Immunohistochemistry

**Fig. 1:**
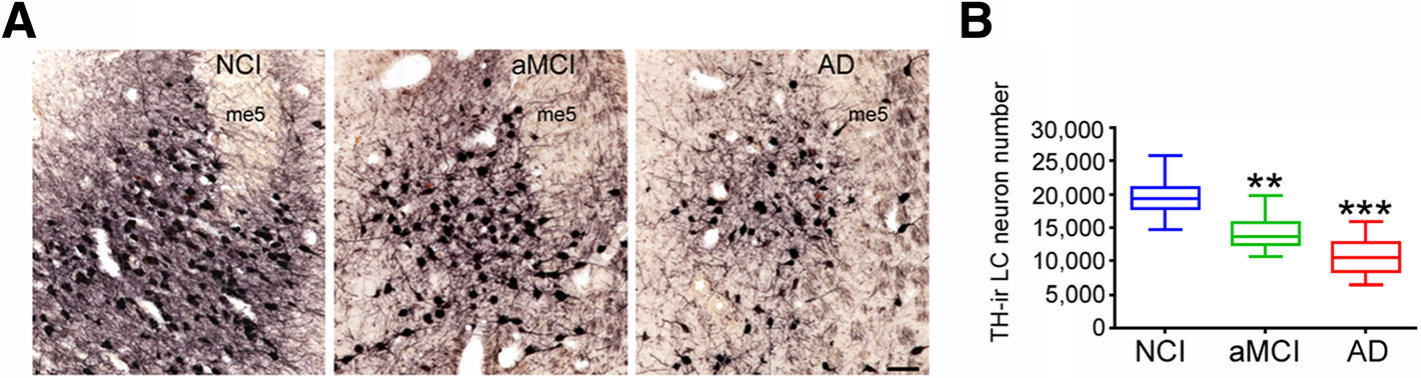
LC cell loss in aMCI. **a** Representative TH-ir neuron and fiber staining in dorsal pontine tissue harvested from NCI, aMCI, and mild/moderate AD subjects. **b** Unbiased stereological cell counts revealed a significant ~30% decrease in the number of LC neurons in aMCI compared to NCI cases. There was a ~50% loss of LC neurons in the AD group compared to NCI. **, *p* < 0.01, ***, *p* < 0.001 compared to NCI, via one-way ANOVA with Bonferroni *post hoc* testing. There was also a significant ~25% difference in neuronal cell counts between the aMCI and AD groups (*p* < 0.05). me5 = mesencephalic tract of 5. Scale Bar = 100 μm

Fixed brainstem samples containing the dorsal pons were cut at 40 μm-thickness on a freezing, sliding microtome into 18 adjacent series and stored in a cryoprotectant solution until processed. Sections containing the LC were immunostained for TH to visualize noradrenergic neurons [35, 65]. A full 1:18 series of sections was blocked in TBS/0.25% Triton X-100/10% goat serum and incubated overnight with rabbit TH antiserum (1:500; Millipore, Billerica, MA). The sections were then sequentially incubated with biotinylated goat anti-rabbit IgG and avidin-biotin complex (ABC; Vector Labs, Burlingame, CA) and developed using 3,3′-diaminobenzidine (DAB) enhanced with nickel II sulfate to yield a blue-black reaction product (Fig. 1).

### Stereological analysis of LC number

The optical disector method was used to determine the number of TH-ir neurons in the left hemisphere of the LC (StereoInvestigator, MicroBrightField; Williston, VT) using an Olympus BX-60 microscope coupled to a Prior H128 computer-controlled *x-y-z* motorized stage and a high-sensitivity Hitachi 3CCD video camera system [90, 91]. Previous studies have demonstrated equivalent neuron numbers between the left and right hemispheres of the LC [25, 50, 93]. The region containing the LC and subcoeruleus was outlined at 5x and disectors were placed at 1000 μm steps along the *x*- and *y*-axis from a random start within this reference space. The sampling strategy was optimized using the StereoInvestigator oversample-resample probe, such that TH-ir neurons were counted under a 60x planar oil-immersion objective (1.4 numerical aperture) in a 120 μm^2^ counting frame with a 10 μm dissector height. Once the top of the section was in focus, the *z*-plane was lowered 1–2 μm. Care was taken to ensure that the top forbidden plane was never included in the analysis. Using these parameters, at least 200 TH-ir neurons were sampled from each series, achieving coefficients of error [56] values of <0.10 [118]. In the present study, lightly and darkly stained TH-ir neurons containing either a well-defined nucleus or nucleolus were counted by a stereologist blinded to the age, sex, cause of death, and clinical classification. Antibody penetration analysis through the full depth of the section was performed to ensure that all objects were counted.

### LC neuronal accession and gene expression profiling

TH-labeled tissue sections processed for custom-designed microarray analysis were prepared without cover-slipping and maintained in RNase-free conditions as described previously for cholinergic nucleus basalis neurons and CA1 pyramidal neurons [27, 53, 54, 111]. Approximately 50 TH-ir LC neurons were captured per sample and a total of 3–4 samples of LC neurons/case were accessed by laser capture microdissection (LCM; ArcturusXT; Applied Biosystems, Foster City, CA) and subjected to custom-designed microarray analysis (5150 total neurons, 103 arrays total) [27, 53, 54, 111].

RNA amplification from LC neurons was performed using terminal continuation (TC) RNA amplification [3, 26, 52]. Briefly, microaspirated LC neurons were homogenized in 500 μL Trizol reagent (Invitrogen, Carlsbad, CA). RNAs were reverse transcribed in the presence of the poly d(T) primer (100 ng/μl) and TC primer (100 ng/μl) in 1x first strand buffer (Life Technologies, Carlsbad, CA), 2 μg of linear acrylamide (Applied Biosystems), 10 mM dNTPs, 100 μM DTT, 20 U of SuperRNase Inhibitor (Life Technologies), and 200 U of reverse transcriptase (Superscript III, Life Technologies). Single-stranded cDNAs were digested with RNase H and re-annealed with the primers in a thermal cycler: RNase H digestion step at 37 °C, 30 min; denaturation step 95 °C, 3 min; primer re-annealing step 60 °C, 5 min. This step generated cDNAs with double-stranded regions at the primer interface. Samples were purified by column filtration (Montage PCR filters; Millipore). Hybridization probes were synthesized by in vitro transcription using ^33^P incorporation in 40 mM Tris (pH 7.5), 6 mM MgCl2, 10 mM NaCl, 2 mM spermidine, 10 mM DTT, 2.5 mM ATP, GTP and CTP, 100 μM of cold UTP, 20 U of SuperRNase Inhibitor, 2 KU of T7 RNA polymerase (Epicentre, Madison, WI), and 120 μCi of ^33^P-UTP (Perkin-Elmer, Boston, MA) [3, 27, 53] at 37 °C for 4 h. Radiolabeled TC RNA probes were hybridized to custom-designed microarrays without further purification. Arrays were hybridized overnight at 42 °C in a rotisserie oven and washed sequentially in 2X SSC/0.1% SDS, 1X SSC/0.1% SDS, and 0.5X SSC/0.1% SDS for 20 min each at 42 °C. The arrays were placed in a phosphor screen for 24 h and developed on a Storm phosphor imager (GE Healthcare, Piscataway, NJ).

### Custom-designed microarray platforms and data collection

Array platforms consisted of 1 μg linearized cDNA purified from plasmid preparations adhered to high-density nitrocellulose (Hybond WL, GE Healthcare) [3, 26, 52]. The array platform consisted of approximately 576 cDNAs selected to provide a broad spectrum of markers related to pathways of interest in neurobiology (see [111] for a list of array targets). Hybridization signal intensity was determined using Image Quant software (GE Healthcare) and quantified by subtracting background using an empty vector (pBluescript). Expression of TC amplified RNA bound to each linearized cDNA minus background was expressed as a ratio of the total hybridization signal intensity of the array (i.e., global normalization) [27, 52]. The data analysis generated expression profiles of relative changes in mRNA levels among the noradrenergic LC neurons dissected from each case within the clinical diagnostic groups.

### Data analysis and statistics

Demographic variables (Table 1) were compared among clinical diagnostic groups by Kruskal-Wallis or Fisher’s Exact tests with Bonferroni correction for pairwise comparisons. LC neuron number was compared across groups by one-way ANOVA with Bonferroni *post hoc* testing. Associations between LC neuron number and clinical pathological variables were tested using Spearman rank correlations. Relationships found to be significant by correlation were investigated further using linear regression analysis. The level of statistical significance was set at *p* < 0.05.

A one-way ANOVA with *post hoc* Newman-Keuls analysis was used to evaluate relative changes in total hybridization signal intensity for individual mRNAs. The level of statistical significance was set at *p* < 0.01. A false discovery rate controlling procedure was used to reduce type I errors due to the large number of genes analyzed simultaneously [4, 26, 31, 103]. Expression levels of select mRNAs were clustered and displayed using a bioinformatics and graphics software package (GeneLinker Gold, Improved Outcomes, Kingston, ON). Associations between the expression levels of select transcripts and clinical pathological variables were tested using Spearman rank correlations. The level of statistical significance was set at *p* < 0.05.

## Results

### Case demographics

Table 1 summarizes the clinical, neuropathological, and demographic characteristics of the 29 cases examined. Statistical analysis revealed no significant differences in age, gender, education level, ApoE4 status, or postmortem interval (PMI) across the groups examined. Cognitive testing scores were available within the last year of life. The average interval from last evaluation to time of death was 7.2 ± 2.8 months with no significant differences among the 3 diagnostic groups (*p* = 0.63). The AD subjects performed significantly worse on the MMSE compared to the NCI cases (*p* = 0.0008), and GCS *z*-scores were significantly decreased in aMCI and AD compared to NCI (*p* = 0.0002). Braak scores were also significantly different across the clinical groups. The NCI cases displayed significantly lower Braak scores than the AD group (*p* = 0.02). NCI cases were classified as Braak stages I/II (36%), III/IV (54%), or V/VI (10%). The aMCI cases met the criteria for Braak stages I/II (10%), III/IV (70%), and V/VI (20%), and the AD cohort was classified as either Braak stages III/IV (38%) or V/VI (62%). The NIA-Reagan diagnosis for likelihood of AD significantly differentiated NCI and aMCI from AD subjects (*p* = 0.03). CERAD scores were significantly higher in AD compared to NCI and aMCI (*p* = 0.02).

### LC neuronal cell loss during the progression of AD

TH immunohistochemistry was used to estimate changes in the number of noradrenergic LC neurons across the clinical diagnostic groups (Fig. 1). Qualitatively, we observed a step-wise decrement in TH-ir neurons within the LC from NCI to aMCI to mild/moderate AD (Fig. 1a). Unbiased stereological cell counts validated these observations. The estimated number of TH-ir LC neurons in the NCI group was 19,495 ± 2,891(mean ± SD; range = 25,867–14,758), whereas neuron number was progressively decreased in the aMCI (14,283 ± 2,757; range = 19,874–10,645) and AD ( 10,628 ± 2,946; range = 15,834–6,453) groups. Statistical comparisons revealed a significant ~30% decrease in the number of LC neurons in aMCI compared to NCI cases (*p* < 0.01) (Fig. 1b). An additional ~25% decrease in LC neuron number was quantified in AD compared to aMCI (*p* < 0.05), resulting in a ~45–50% loss of LC neurons in the AD group compared to NCI (*p* < 0.001) (Fig. 1b). Total LC neuron number was similar between males and females in each diagnostic group (data not shown).

### Clinical pathologic correlations

**Fig. 2:**
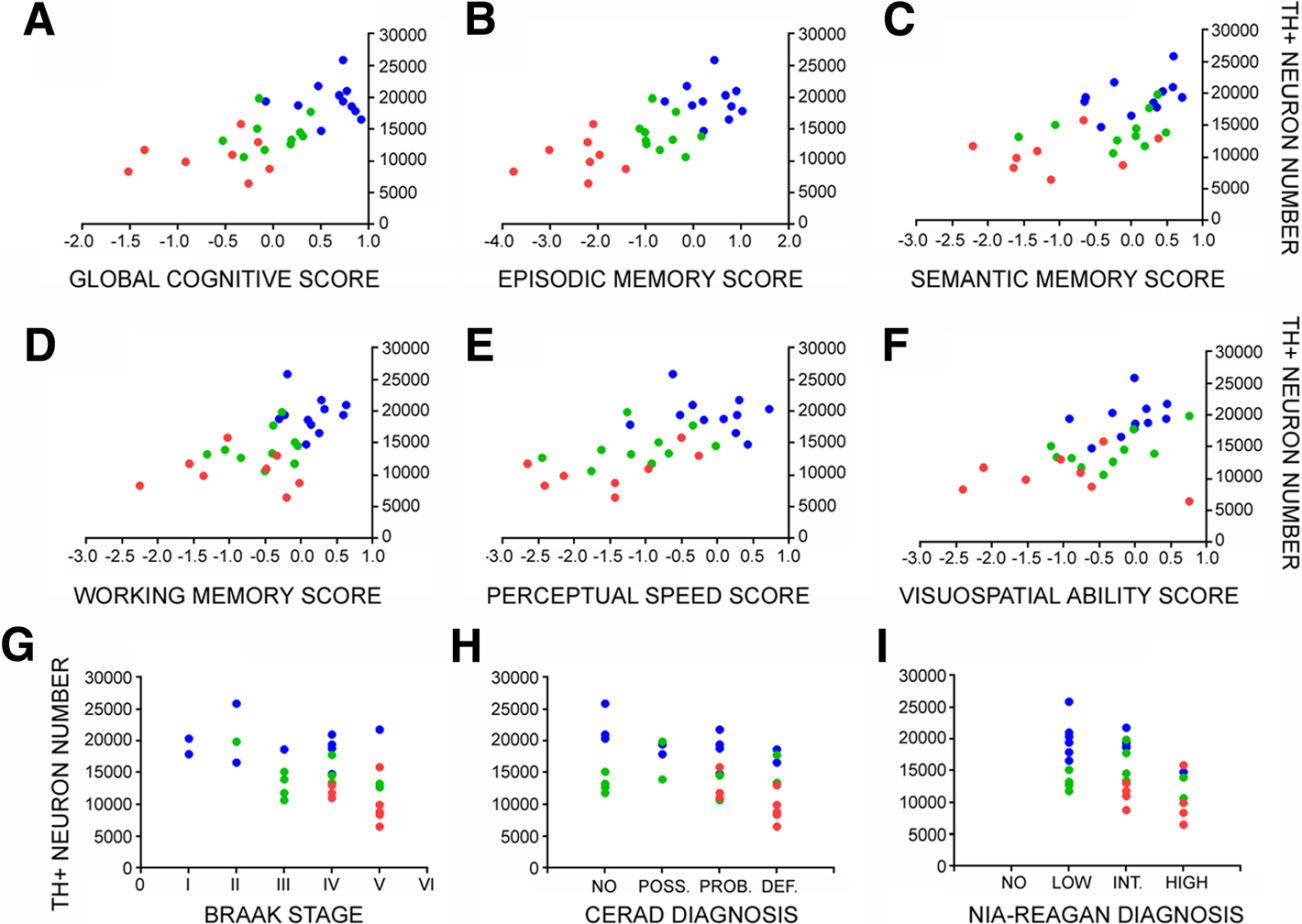
TH-ir LC neuron number correlates with multiple measures of antemortem cognition and postmortem neuropathology during the progression of AD. Scatterplots show significant relationships between reductions in LC neurons and **a** the GCS for each individual (*r* = 0.7, *p* < 0.0001), as well as poorer performance on composite scores of **b** episodic memory (*r* = 0.71, *p* < 0.0001), **c** semantic memory (*r* = 0.598, *p* = 0.0008), **d** working memory (*r* = 0.6, *p* = 0.0006), **e** perceptual speed (*r* = 0.66, *p* < 0.0001), and **f** visuospatial ability (*r* = 0.53, *p* = 0.003). There were also significant negative correlations between TH+ neuron number and increasing neuropathology as defined by **g** Braak (*r* = −0.46, *p* = 0.01), **h** CERAD (*r* = −0.41, *p* = 0.03), and **i** NIA-Reagan (*r* = −0.45, *p* = 0.01) diagnostic criteria. All associations were tested using Spearman rank correlation analysis. Abbreviations: TH+ (TH-positive), POSS. (possible), PROB. (probable), DEF. (definite), INT. (intermediate). Symbols: NCI (*blue-filled circle*), aMCI (*green-filled circle*), AD (*red-filled circle*)

Estimated TH-ir LC neuronal counts were correlated with demographic data, antemortem cognitive test performance, and postmortem neuropathologic variables. Neuron number was not associated with age (*r* = −0.18, *p* = 0.3) or PMI (*r* = 0.07, *p* = 0.5). There were no associations between neuron number and gender or ApoE status (data not shown). However, there was a strong association between reduction in LC neuron number and decline in cognitive status. For instance, lower LC neuron numbers and MMSE scores displayed a significant positive association (*r* = 0.61, *p* < 0.001). Moreover, TH-ir LC neuron number was significantly associated with individual GCS, the composite *z*-score of the 17 neuropsychological tests administered prior to death (*r* = 0.7, *p* < 0.0001) (Fig. 2), as well as with the final composite scores referable to episodic memory (*r* = 0.71, *p* < 0.0001), sematic memory (*r* = 0.58, *p* = 0.0008), working memory (*r* = 0.6, *p* = 0.0006), perceptual speed (*r* = 0.66, *p* < 0.0001), and visuospatial ability (*r* = 0.53, *p* = 0.003; Fig. 2) [14]. Notably, most of these positive associations between LC neuron numbers and cognitive test scores remained significant even when the NCI and aMCI groups alone were analyzed (Table 2). With respect to neuropathological diagnostic criteria, reductions in TH-ir LC neuron numbers were negatively correlated with increasing measures of neuropathology as characterized by Braak (*r* = −0.46, *p* = 0.01), NIA-Reagan (*r* = −0.45, *p* = 0.01), and CERAD (*r* = −0.41, *p* = 0.03) staging (Fig. 2). Finally, regression modeling revealed that only GCS (*p* = 0.004), but not Braak (*p* = 0.49), NIA-Reagan (*p* = 0.81), or CERAD (*p* = 0.28) score, served as a predictor for the number of TH-ir LC neurons.

**Table 2:** Correlations between neuron number and cognitive test scores: NCI and aMCI subjects only

MMSE	*r* = 0.46 (*p* = 0.03)
GCS	*r* = 0.58 (*p* = 0.006)
episodic memory	*r* = 0.55 (*p* = 0.01)
semantic memory	*r* = 0.29 (*p* = 0.2)
working memory	*r* = 0.6 (*p* = 0.004)
perceptual speed	*r* = 0.51 (*p* = 0.02)
visuospatial ability	*r* = 0.55 (*p* = 0.009)

### Dysregulation of LC neuronal gene expression in MCI and AD

**Fig. 3:**
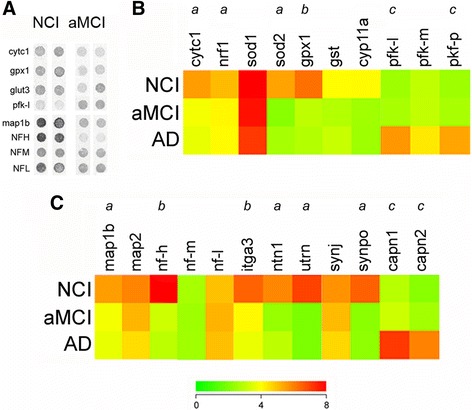
Single LC neuron expression profiling reveals alterations in mRNAs regulating mitochondrial and neuritic function during AD progression. **a** Primary custom microarray data showing hybridization signal intensities of select mRNA transcripts within TH-ir LC neurons in NCI and aMCI. Abbreviations: cytochrome C1 (*Cytc1*), glutathione peroxidase (*Gpx1*), glucose transporter 3 (*Glut3*), phosphofructokinase-liver isozyme (*Pfkl*), microtubule associated binding protein 1a (*Map1a*), and neurofilament heavy (*Nfh*), medium (*Nfm*), or light (*Nfl*) chain subunits. **b** Heatmap shows significant decreases in transcripts regulating mitochondrial fucntion in LC neurons in aMCI and AD cases compared to NCI, including *Cytc1*, nuclear respiratory factor 1 (*Nrf1*), superoxide dismutase 2 (*Sod2*) and *Gpx1*, whereas *Pfkl* and platelet (*Pfkp*) isozymes were up-regulated in mild AD (red to green = decreasing expression levels). Additional abbreviations: superoxide dismutase 1 (*Sod1*), glutathione transferase (*Gst*), cytochrome p450 11a (*Cyp11a*), and pfk-muscle (*Pfkm*). **c** Heatmap shows significant decreases in transcripts regulating cytoskeletal/structural plasticity in LC neurons in aMCI and AD cases compared to NCI, including *Map1b*, *Nfh*, integrin 3 (*Itga3*), netrin 1 (*Ntn1*), utrophin (*Utrn*), and synaptopodin (*Synpo*), whereas calpain 1 (*Capn1*, m-calpain) and calpain 2 (*Capn2*, μ-calpain) were up-regulated in mild AD (red to green = decreasing expression levels). Additional mRNAs: mitochondrial associated protein 2 (*Map2*), and synaptojanin (*Synj*). *a*, NCI > aMCI, AD, *p* < 0.01; *b*, NCI > aMCI, AD, *p* < 0.001; *c*, AD > NCI, aMCI, *p* < 0.01

To identify potential molecular pathogenic alterations underlying LC cell loss observed during AD progression, we performed custom-designed microarray analysis using mRNA extracted from individual TH-ir LC neurons accessed from the NCI, aMCI and mild/moderate AD cases. LC neurons displayed a significant downregulation of transcripts regulating mitochondrial function in aMCI and AD compared to NCI subjects (Fig. 3a, b). Significant downregulation was observed for select genes regulating respiration, including cytochrome C1 (*Cytc1*; 50%; *p* < 0.01) and nuclear respiratory factor 1(*Nrf1*; 45%; *p* < 0.01), as well as redox gene expression, including superoxide dismutase 2 (*Sod2*; 50%; *p* < 0.01) and glutathione peroxidase 1 (*Gpx1*; 55%; *p* < 0.001). By contrast, significant upregulation in the expression of the phosphofructokinase-liver (*Pfkl*; 50%; *p* < 0.01) and phosphofructokinase-platelet (*Pfkp*; 50%; *p* < 0.01) isozymes were detected in LC neurons from AD subjects relative to NCI and aMCI (Fig. 3a, b).

Gene expression analysis also revealed a significant downregulation of transcripts related to neuritic/structural plasticity in cases with an antemortem clinical diagnosis of aMCI and AD compared to NCI, including mRNAs encoding microtubule associated binding protein 1b (*Map1b*; 50%; *p* < 0.01), neurofilament heavy chain (*Nfh*; 65%; *p* < 0.001), cell surface adhesion molecule integrin alpha 3 subunit (*Itga3*; 55%; *p* < 0.001), axon guidance protein netrin (*Ntn1*; 40%; *p* < 0.01), postsynaptic clustering protein utrophin (*Utrn*; 45%; *p* < 0.01), and the dendritic spine plasticity protein synaptopodin (*Synpo*; 60%; *p* < 0.01) (Fig. 3a, c). Moreover, an analysis of transcripts encoding the six tau isoforms (*Mapt1*-*Mapt6*) [54] revealed a significant ~25–30% decrease in the ratio of 3-repeat tau (3Rtau) to 4-repeat tau (4Rtau) isoform expression within single LC neurons in aMCI and AD compared to NCI cases (Table 3). In contrast, an upregulation of the cytoskeletal proteases calpain 1 (*Capn1*; 45%; *p* < 0.01) and calpain 2 (*Capn2*; 55%; *p* < 0.01) was found in AD (Fig. 3c).

**Table 3:** 3Rtau/4Rtau mRNA ratios in single LC neurons

Clinical diagnosis	3R/4R ratio 0 insert	3R/4R ratio 1 insert	3R/4R ratio 2 inserts
NCI	1.36 ± 0.11	1.12 ± 0.14	1.25 ± 0.07
aMCI	1.02 ± 0.09*	0.81 ± 0.08*	0.92 ± 0.12*
AD	0.98 ± 0.1*	0.83 ± 0.05*	0.89 ± 0.13*

* *p* < 0.05 via one-way ANOVA with Bonferroni correction

Several transcripts encoding other functional classes of genes relevant to AD pathogenesis were unaffected. For example, levels of amyloid-β precursor protein (*App*) and several genes related to APP metabolism, such as beta-site APP-cleaving enzyme 1 (*Bace1*), presenilin 1 (*Psen1*), and APP family member genes amyloid precursor-like protein 1 (*Aplp1*) and amyloid precursor-like protein 2 (*Aplp2*) were not differentially regulated in LC neurons across the clinical groups (Additional file 1: Figure S1).

### Correlations with clinical pathologic variables

**Fig. 4:**
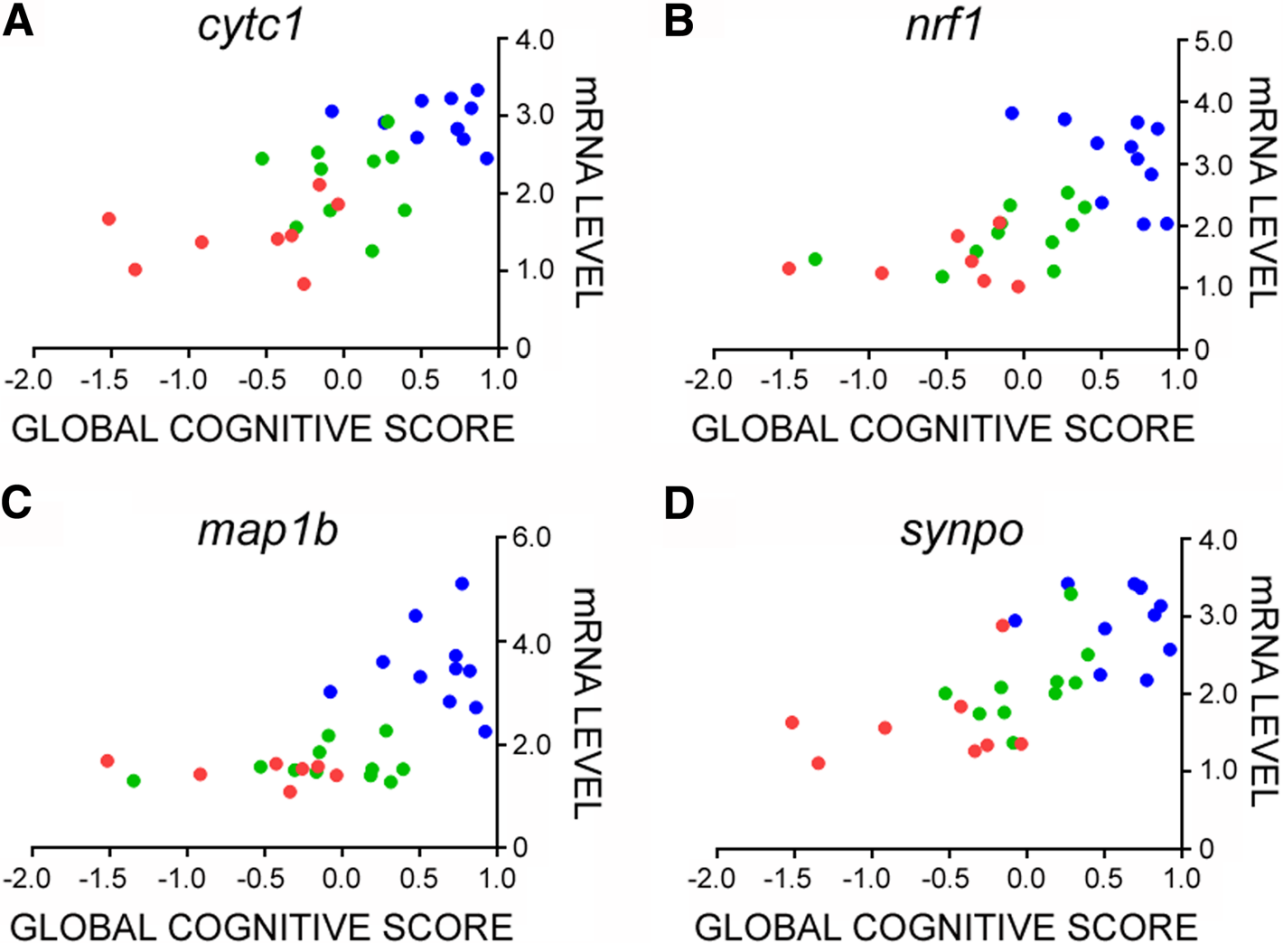
Alterations in select transcripts isolated from noradrenergic LC neurons correlate with global cognition during the progression of AD. Scatterplots show significant relationships between decreasing levels of **a** *Cytc1* (*r* = 0.64, *p* < 0.0001), **b** *Nrf1* (*r* = 0.57, *p* = 0.0003), **c** *Map1b* (*r* = 0.59, *p* = 0.0006), and **d** *Synpo* (*r* = 0.65, *p* < 0.0001) and worsening GCS score, via Spearman rank correlation analysis. Symbols: NCI (*blue-filled circle*), aMCI (*green-filled circle*), AD (*red-filled circle*)

Although there were no associations between transcript levels and age or PMI, there was a strong relationship between downregulation of LC metabolic and structural plasticity gene expression and decreased GCS performance (Fig. 4) [14]. The four strongest associations were between GCS and *Cytc1* (*p* < 0.0001, Fig. 4a), *Nrf1* (*p* = 0.0003, Fig. 4b), *Map1b* (*p* = 0.0006, Fig. 4c) and *Synpo* (*p* < 0.0001, Fig. 4d) transcripts. There were also significant associations between metabolic and structural plasticity gene expression and MMSE scores (Table 4). By comparison, stable levels of superoxide dismutase 1 (*Sod1*; Fig. 3b) were not associated with GCS or MMSE scores across diagnostic groups. Virtually all of these select transcript reductions correlated negatively with increasing measures of neuropathology (Braak, NIA-Reagan, and CERAD diagnostic criteria; Table 4).

**Table 4:** Clinical pathologic correlations of select LC neuronal transcripts dysregulated in aMCI and AD

transcript	GCS	MMSE	Braak	NIA-Reagan	CERAD
*cytc1*	*r* = 0.64 (*p* < 0.0001)	*r* = 0.48 (*p* = 0.003)	*r* = −0.50 (*p* = 0.003)	*r* = −0.41 (*p* = 0.02)	*r* = −0.48 (*p* = 0.01)
*nrf1*	*r* = 0.57 (*p* = 0.0003)	*r* = 0.37 (*p* = 0.04)	*r* = −0.43 (*p* = 0.08)	*r* = −0.39 (*p* = 0.06)	*r* = −0.50 (*p* = 0.003)
*gpx1*	*r* = 0.54 (*p* = 0.008)	*r* = 0.44 (*p* = 0.008)	*r* = −0.42 (*p* = 0.02)	*r* = −0.42 (*p* = 0.01)	*r* = −0.39 (*p* = 0.02)
*map1b*	*r* = 0.59 (*p* = 0.0006)	*r* = 0.35 (*p* = 0.03)	*r* = −0.43 (*p* = 0.01)	*r* = −0.33 (*p* = 0.2)	*r* = −0.32 (*p* = 0.09)
*nf-h*	*r* = 0.54 (*p* = 0.0007)	*r* = 0.51 (*p* = 0.001)	*r* = −0.55 (*p* < 0.001)	*r* = −0.49 (*p* = 0.005)	*r* = −0.45 (*p* = 0.006)
*ntn1*	*r* = 0.52 (*p* = 0.0008)	*r* = 0.44 (*p* = 0.007)	*r* = −0.41 (*p* < 0.01)	*r* = −0.40 (*p* = 0.02)	*r* = −0.47 (*p* = 0.006)
*synpo*	*r* = 0.65 (*p* < 0.0001)	*r* = 0.52 (*p* < 0.001)	*r* = −0.58 (*p* < 0.001)	*r* = −0.47 (*p* = 0.008)	*r* = −0.45 (*p* = 0.005)
*sod1*	*r* = 0.22 (*p* = 0.1)	*r* = −0.11 (*p* = 0.5)	*r* = −0.18 (*p* = 0.6)	*r* = −0.09 (*p* = 0.6)	*r* = −0.28 (*p* = 0.1)

### Microarray validation

Since all brainstem tissue containing the LC from the RROS cohort is immersion-fixed [106, 120], qPCR and immunoblot validation analyses of frozen tissue samples were not conducted as previously described [4, 27, 53]. However, changes associated with the noradrenergic phenotype at the single neuron level would likely be masked by regional expression patterns from admixed neuronal and non-neuronal cell types included in the frozen samples required for these analyses, similar to our observations in cholinergic neurons in the nucleus basalis/substantia innominata [28, 30, 54]. On the other hand, studies using regional gene expression analysis or neuronal immunohistochemical analysis in vulnerable brain regions have reported findings that support our LCM-based custom microarray approach. For instance, *Map1b* gene expression was downregulated in AD temporal cortex [123], whereas downregulation of Synpo protein in dendritic spines occurred in frontal cortex neurons in AD [9] similar to that found in our LC gene array experiments. Future studies employing single population RNA-sequencing, Fluidigm, and/or Nanostring nCounter analyses are warranted when transcriptomic technologies become more standardized and economical [22, 66, 73].

## Discussion

We demonstrate in this report that TH-ir, presumably noradrenergic LC neurons are vulnerable during the onset of dementia as evidenced by their loss in aMCI and AD and the association of this loss with multiple measures of cognitive deterioration and neuropathological accumulation. We further characterized the molecular underpinnings of LC neuronal vulnerability by single population microarray analysis. Results indicated selective changes in genes regulating mitochondrial function and neuritic/structural plasticity, which also correlated with antemortem cognitive status and postmortem plaque and tangle burden. Hence, the early loss of forebrain monoaminergic signaling mediated by the LC appears to be central to AD pathophysiology.

Rodent and non-human primate investigations have revealed the importance of the ascending noradrenergic LC projection system for cognitive function [11, 16, 85]. Based on a series of seminal experiments, Aston-Jones and Cohen proposed a theory of ‘adaptive gain’ whereby NE integrates sensory, attentional and memory processing by positively modulating signal gain in neurons to facilitate the processing of salient events by driving LC phasic activity in response to relevant stimuli [11]. LC bursts and pulsatile NE efflux in coeruleus target fields (e.g., hippocampus and prefrontal cortex) increases the activity of excitatory inputs and decreases the action of inhibitory inputs, which optimizes task performance [11, 16]. Despite the more caudal location of the LC deep within the brainstem it might be envisioned as the brain’s “watchtower,” scanning incoming sensory information for important events that require immediate attention [33]. Moreover, NE-dependent modulation of long-term alterations in synaptic strength and gene transcription, particularly within the hippocampus and prefrontal cortex, influences memory formation and experience-dependent alterations in neural function and behavior and plays a critical role in the ability of the LC to optimize performance [105]. Finally, evidence from transgenic mouse models of AD and neuronal cell culture studies show that NE exerts a wide array of neuroprotective effects including anti-inflammatory and pro-neurotrophic mechanisms [32, 43, 44, 58, 64, 72, 74, 117]. Taken together, these data suggest that the central NE projection system is essential for cognitive function and, in turn, that LC neuronal degeneration contributes to cognitive dysfunction.

Recently, two reports have shown that LC neurodegeneration coincides with both mounting Braak stage pathology [110] and cognitive impairment [7]. Theofilas and colleagues reported that LC volume decreases ~8.4% with each successive Braak stage, resulting in a significant ~25% loss of LC volume between control cases neuropathologically diagnosed postmortem as Braak stage III compared to stage 0 [110]. This volume loss mirrored a similar rate of change in total pigmented and non-pigmented LC neuron numbers, as measured by unbiased stereology [110]. The authors also noted that the topography of LC volume and cell loss appears to follow a rostrocaudal gradient similar to that reported in frank AD cases [49, 110]. Here, we report that the loss of TH-ir, presumably noradrenergic LC neurons also correlates with Braak stage. In addition, we found that TH-ir neuron loss correlated with increasing neuropathologic burden based on NIA-Reagan and CERAD diagnostic criteria. As reductions in LC neuron number have been associated with increased cortical amyloid plaque and NFT loads in cases of frank AD [18, 108], these observations in early stage cases indicate a strong relationship between LC projection system degeneration and the pathologic sequela of AD.

Arendt and colleagues used unbiased stereology to demonstrate a significant ~13% loss of neuromelanin-positive LC neurons in subjects classified as MCI/prodromal AD (CDR 0.5 who also displayed “low” to “intermediate” amyloid-Braak-CERAD (ABC) diagnostic scores [61]) compared to those classified as controls (CDR 0 and “not” ABC score) [7]. Subjects classified as mild/moderate AD exhibited ~30–45% LC cell loss compared to controls. This study revealed that LC cell loss, which is prominent in cases of frank AD [19, 24, 36, 75, 86, 119], appears to occur early in the clinical progression of AD, concurrent with cell loss in the nucleus basalis and entorhinal cortex [7].

The present morphometric analysis revealed a ~30% loss of TH-ir LC number during the transition from NCI to aMCI in cases classified independent of neuropathological diagnosis. Notably, our estimate of total TH-ir LC cell number in NCI/healthy controls (19,495 ± 2,891) was similar to other studies [23, 50, 93, 112] and, more specifically, to Arendt and colleagues’ unbiased estimate of total neuromelanin-positive neurons (17, 487 ± 2,736) for this group. The discrepancy in the observed loss of LC neurons between control and MCI/prodromal AD between these two studies (~30% compared to ~13%) could be related to cohort group classifications or possibly to a relatively greater loss of TH-expressing neurons compared to neuromelanin-positive neurons early in the disease process. In this regard, it would be interesting to determine whether a subset of neuromelanin-positive LC neurons undergoes a phenotypic downregulation of TH during the disease. Regardless, the present findings suggest that a disconnection of transmitter-identified LC projections to the forebrain contributes to the presentation of clinical disease.

With respect to the relationship between LC degeneration and cognitive status across the diagnostic groups, we found that reduced numbers of TH-ir LC neurons were associated not only with episodic memory deficits that define aMCI subtypes [63], but also with semantic memory, working memory, perceptual speed, and visuospatial ability. Since the LC innervates components of the dorsal memory network (e.g., dorsolateral and dorsomedial prefrontal cortex) [10, 84] and the amygdala [46, 99, 115], which are both dysfunctional early in the onset of AD [89], disruption of NE afferents to these structures may play a role in the deficits seen in these various cognitive domains [14]. In fact, we found that quantitative decreases in LC neuron number were associated with poorer performance on measures of global cognitive function, as well. Hence, we demonstrate a link between loss of noradrenergic tone, neuropathological criteria, and cognitive decline. However, regression analysis revealed that global cognitive decline, but not neuropathological status, was a predictor for LC cell number. These findings lend support to a previous autopsy study, which showed that higher LC neuronal density was associated with slower rates of antemortem cognitive decline, suggesting a role for the LC in cognitive reserve [120]. Altogether, our data supports an emerging theory that LC neuron degeneration is an early pathological event that contributes to cognitive dysfunction and the onset of AD, and is likely a site for therapeutic intervention.

The cellular and molecular mechanisms underlying LC selective vulnerability in the early stages of AD are unclear. To begin to address this question, we profiled gene expression patterns of transmitter-identified LC neurons microdissected from NCI, aMCI, and AD cases. We noted two major patterns of expression changes related to functional classes of genes regulating mitochondrial function and neuritic plasticity. With respect to the transcripts involved in mitochondrial dysfunction, *Nrf1* and *Cytc1* mRNA levels were both significantly downregulated in LC neurons in aMCI and mild AD relative to NCI. Nrf1 is a transcription factor that directs the expression of several functional classes of genes involved in mitochondrial function, including those regulating redox homeostasis, mitochondrial biogenesis, calcium homeostasis, and cytochrome oxidase activity [17, 40, 41]. Interestingly, Cytc1 is the heme-containing component in the cytochrome b-c1 complex III of the respiratory chain, accepting electrons from Rieske protein and transferring it to cytochrome c, which couples to cytochrome oxidase [47, 113]. By contrast, transcript levels of *Pfkl* and *Pfkp* isozymes were significantly upregulated in LC neurons in AD. These enzyme subunits catalyze the conversion of D-fructose 6-phosphate to D-fructose 1,6-bisphosphate, resulting in the first committing step of glycolysis [60]. Taken together, these results indicate that LC neurons are under considerable respiratory stress during the transition from NCI to prodromal and frank AD. In addition, these neurons displayed significant decreases in transcripts encoding the antioxidant enzymes mitochondrial Sod2 and Gpx1 in aMCI. Given the role of these two enzymes in detoxifying superoxide and hydrogen peroxide, respectively, LC neurons are also likely under considerable oxidative stress prior to the onset of aMCI [109]. These observations support tissue-based assessments demonstrating regional mitochondrial deficits during the progression of AD [12, 15, 59, 71, 101, 102, 116, 125], but importantly, identify these changes in neurons, as opposed to glial cells or admixed populations of cells, thus attributing these markers for mitochondrial dysfunction to LC neurons.

A second common thread of transcript dysregulation in aMCI centered on genes encoding proteins involved in axonal function and neuronal morphological plasticity. For instance, Map1b is critical for microtubule stabilization during axonal growth [114] and Ntn1 plays a role in axonal guidance [81], whereas Nfh is essential in maintaining axonal caliber and is dysregulated in human AD and animal models of the disease [42, 83]. Significant downregulation of these genes suggest that LC neurons may be undergoing an axonal degenerative process during prodromal stages, which progress to frank AD as evidenced by a significant increase in the cytoskeletal proteases Capn1 and Capn2 expression in AD neurons [92, 104]. Moreover, we found a global decrease in the ratio of 3R/4R tau isoforms in LC neurons in aMCI and AD, consistent with previous single cell gene expression studies of cholinergic nucleus basalis and CA1 pyramidal neurons isolated from MCI and AD cases [54]. The alteration in 3R tau gene dosage relative to 4R tau suggests a shift toward tau isoforms associated with slower axonal transport kinetics as well as NFT formation [37, 69, 107].

Finally, we observed a concomitant downregulation of genes encoding the postsynaptic receptor clustering protein Utrn and the dendritic spine marker Synpo in aMCI and AD neurons compared to NCI. In particular, Synpo is an actin-associated protein that may play a role in modulating actin-based shape and motility of dendritic spines and seems to be essential for the formation of spine apparatuses involved in synaptic plasticity [39, 68]. Dysregulation of postsynaptic gene expression in vulnerable LC neurons may signal perturbations prior to the onset of aMCI that are indicative of a failure in neuroplastic and/or remodeling programs [38, 39, 68, 89]. While we cannot rule out the possibility that these molecular changes are epiphenomenal to AD pathogenesis, the timing of these changes to stages prior to the onset of aMCI suggest that they represent pathways critical to disease progression.

Perhaps the most striking feature of the LC is the immensity and divergence of its noradrenergic forebrain efferents [45], extensive complex dendritic arborization pattern, and the long distance these perikarya project to reach their forebrain innervation sites [6, 8, 85]. In this regard, LC neurons are similar to other selectively vulnerable long forebrain projection neuron systems (e.g., cholinergic basal forebrain neurons, substantia nigra pars compacta neurons, and dorsal raphe neurons, among others [5, 16, 78, 80, 105]), that are heavily reliant on energy metabolism and cytoskeletal integrity to modulate synaptic input. These long projection systems are likely more prone to cellular stress given their position in the brain’s organization. Taken together, our findings present evidence for the molecular dysregulation of mitochondrial function and neuritic/structural plasticity coinciding with the loss of LC neurons prior to the transition from NCI to prodromal AD. Moreover, our observation that the dysregulation of these genes correlates with poorer global cognition and greater neuropathological burden suggests that these molecular pathways represent pathogenic mechanisms underlying the selective vulnerability of LC neurons which may be common to other vulnerable neurons. Notably, there is evidence from similar single neuron expression analysis studies showing alterations in mitochondrial and structural plasticity pathways in AD. For instance, we have demonstrated that cholinergic nucleus basalis neurons exhibit alterations in *Sod2*, *Pfkl*, and *Capn1* in AD [29]. Other studies have also demonstrated an AD-related dysregulation of specific respiratory chain genes (e.g., cytochrome oxidase 5b) in medial temporal and posterior cingulate pyramidal neurons [70, 71]. However, the extent to which dysregulation of these pathways are evident in other selectively vulnerable cell groups during the onset of cognitive decline warrants further comparative assessments of these neuron groups as well as relatively unaffected populations in postmortem brain tissues. Moreover, whether dysregulation of these pathways is related to NFT or Lewy body pathology in these neurons is an additional question to be addressed in future studies [51, 55, 62, 111, 112]. The current findings also suggest the need for additional quantitative biochemical and immunohistochemical assessments of these mitochondrial and structural markers in LC neurons in aMCI.

## Conclusions

The present findings point to the continuing need to consider noradrenergic system pathophysiology as a key and early component associated with the progression of AD. We posit that strategies aimed at LC neuroprotection or NE replacement are viable therapeutic options [98]. Moreover, as prominent LC degeneration is also evident in cases of Parkinson’s disease with dementia and dementia with Lewy bodies [57], maintaining LC projection system integrity may provide a common therapeutic mechanism for combating cognitive decline in multiple late-onset dementia subtypes. Here, we present several candidate molecular pathways that are dysregulated in LC neurons early in the cascade of pathogenic events prior to the onset of AD, which may form the basis for novel neuroprotective approaches for dementia.

## Additional file

Additional file 1: Figure S1. No change in AD-related gene expression in LC neurons during disease progression. Bar graph shows changes in *App*, *Bace1*, *Psen1*, *Aplp1*, and *Aplp2* transcripts in aMCI and AD relative to NCI. No significant differences were calculated across the diagnostic groups. (TIF 998 kb)
