# Supplementary figures and images for: Locus coeruleus cellular and molecular pathology during the progression of Alzheimer’s disease
Source: Acta Neuropathol Commun. 2017 Jan 21;5:8. doi: 10.1186/s40478-017-0411-2 (PMC5251221; doi:10.1186/s40478-017-0411-2)

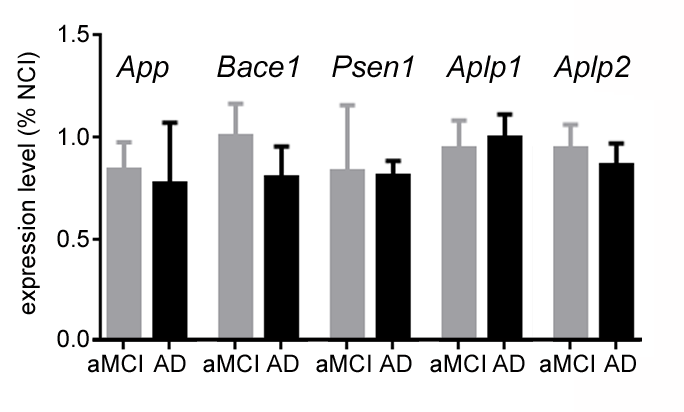

Supplement: Additional file 1: Figure S1. — No change in AD-related gene expression in LC neurons during disease progression. Bar graph shows changes in App, Bace1, Psen1, Aplp1, and Aplp2 transcripts in aMCI and AD relative to NCI. No significant differences were calculated across the diagnostic groups. (TIF 998 kb) [file 40478_2017_411_MOESM1_ESM.tif]
